# What is the Link Between Mental Imagery and Sensory Sensitivity? Insights from Aphantasia

**DOI:** 10.1177/03010066211042186

**Published:** 2021-08-31

**Authors:** C. J. Dance, J. Ward, J. Simner

**Affiliations:** School of Psychology, 1948 University of Sussex, Brighton, UK

**Keywords:** aphantasia, imagery, sensitivity, pattern glare task, dysikonesia, autism traits

## Abstract

People with *aphantasia* have impoverished visual imagery so struggle to form mental pictures in the mind's eye. By testing people with and without aphantasia, we investigate the relationship between sensory imagery and sensory sensitivity (i.e., hyper- or hypo-reactivity to incoming signals through the sense organs). In Experiment 1 we first show that people with aphantasia report impaired imagery across *multiple* domains (e.g., olfactory, gustatory etc.) rather than simply vision. Importantly, we also show that imagery is related to sensory sensitivity: aphantasics reported not only lower imagery, but also lower sensory sensitivity. In Experiment 2, we showed a similar relationship between imagery and sensitivity in the general population. Finally, in Experiment 3 we found behavioural corroboration in a Pattern Glare Task, in which aphantasics experienced less visual discomfort and fewer visual distortions typically associated with sensory sensitivity. Our results suggest for the very first time that sensory imagery and sensory sensitivity are related, and that aphantasics are characterised by both lower imagery, and lower sensitivity. Our results also suggest that aphantasia (absence of *visual* imagery) may be more accurately defined as a subtype of a broader imagery deficit we name *dysikonesia*, in which weak or absent imagery occurs across multiple senses.

Mental imagery is the mechanism by which we mentally simulate perceptual experiences – from visualising a friend's face in the mind's eye, to hearing our favourite song in the ‘mind's ear’ (and similarly for our other senses). Mental images themselves are “iconic” in that visual images in some way resemble internal pictures, and auditory images in some way resemble internal sounds. Being able to form mental images is an essential part of life for many people but this capability varies from person to person. For some people, visual mental imagery is exceptionally strong and nearly as vivid as real-life perception, while for others it is virtually or completely absent, a condition known as *aphantasia* (Zeman et al., 2015, 2016, 2020). One question we ask in this paper is whether the imagery deficit that characterises aphantasia in the visual domain is apparent too in other sense domains. For example, we ask whether people with aphantasia also have impaired imagery for taste, smell, touch and so on. But as well as differing on how sensory information is imaged, people also vary on how sensory information makes them *feel*. Some people have a comfortable tolerance for incoming sensory stimuli from the outside world, while others have *sensory sensitivities* (i.e., an under- or over- responsiveness to sounds, smells, tastes etc., see below; e.g., Robertson and Simmons, 2013). Here, we also ask for the very first time whether sensory sensitivities and sensory imagery are related. Using self-report and behavioural methods, we will show that aphantasic individuals experience not only lower imagery, but also lower sensory sensitivity. We present three experiments testing these ideas but begin with brief overviews of aphantasia, imagery, and sensory sensitivity.

People with aphantasia report either a complete lack of visual mental imagery, or imagery that is only dim, vague or fleeting (e.g., Keogh & Pearson, 2018; Zeman et al., 2015). Aphantasia has until now been defined exclusively as an absence of *visual* imagery (Milton et al., 2021; Zeman et al., 2015, 2016, 2020) but of course mental imagery itself can encompass other modalities too, including auditory, olfactory, gustatory, tactile, motor, and bodily imagery. For us to investigate the nature of aphantasia (and subsequently, to investigate how imagery and sensation are themselves linked) an important initial question is whether the imagery impairment seen in aphantasia also extends to other sense domains. There are a number of reasons to think this might be the case. ‘Aphantasia-like’ deficits in non-visual imagery do indeed exist: there are vast individual differences in the vividness of auditory imagery (Berger & Ehrsson, 2017), olfactory imagery (Leclerc et al., 2019), gustatory imagery (Tiggemann & Kemps, 2005), tactile imagery (Belardinelli et al., 2009), motor imagery (Isaac & Marks, 1994), and bodily imagery (Andrade et al., 2014), with some individuals reporting imagery almost as vivid as real-life perception, while others report a total absence of imagery. Although poor imagery in one domain does not *necessarily* preclude high imagery in another (Andrade et al., 2014), positive correlations in imagery strength do exist across different sensory modalities (Andrade et al., 2014; Leclerc et al., 2019; Lima et al., 2015; White et al., 1974). This suggests that people with aphantasia may indeed have weak/absent imagery in other non-visual domains.

Other suggestions come from first-hand reports from writers with aphantasia (e.g., Watkins, 2018) and from one valuable recent survey of 2,000 aphantasic people, of whom 54% suggested they might have weak/absent imagery in *all* sensory modalities (Zeman et al., 2020). However, this was elicited by a single question (‘*Are all types of imagery affected, or can you imagine sounds (including music), textures (by imagined touch), tastes or smells?*’; Zeman et al., 2020), which required participants to self-diagnose without other means to determine whether their imagery (e.g., gustatory imagery) was better or worse than the average person. A recent study, too, by Dawes et al. (2020) found that aphantasics reported significantly weaker mental imagery than controls in the auditory, tactile, kinaesthetic, taste, olfactory and bodily subscales of Sheehan's adaption of *Bett's Questionnaire Upon Mental Imagery* (Sheehan, 1967). Although their findings represent an important step in understanding the phenomenology of aphantasia, their questionnaire has been criticised for having a small number of items per modality, and for including items that are unclear or out-dated (e.g., imagining ‘the whistle of a locomotive’; see e.g., Pecher et al., 2009; Spiller et al., 2015). Here, we therefore address this same question, measuring aphantasics’ mental imagery across multiple sense domains, but using a series of contemporary standardised questionnaires. These measures may indeed show that the phenomenology of aphantasia extends across multiple senses. Such a finding would be important because it would indicate that the current literature-wide focus on aphantasia as a visual deficit may be hiding a wider phenomenology.

A final reason to suspect that aphantasics may have low or absent imagery in non-visual domains comes from considering its possible neural basis. Visual imagery has been linked to levels of excitability in the visual cortex, meaning that aphantasics may potentially have excitability differences (Cattaneo et al., 2011; Sparing et al., 2002, but see Keogh et al., 2020). If this is true, then these differences in excitability might potentially be found in other sensory regions within the same brain. One way to understand the link between imagery and excitability is to look at studies of the visual cortex using transcranial magnetic stimulation (TMS). Using TMS can induce visual effects known as phosphenes. Importantly, engaging in visual imagery lowers phosphene thresholds (Cattaneo et al., 2011; Sparing et al., 2002), where low thresholds represent high excitability (i.e., less stimulation to generate a phosphene; e.g., Stewart et al., 2001). This suggests that since aphantasics have poor imagery, they may therefore have low excitability. Although phosphene thresholds have not been measured in aphantasia, supporting evidence is that *high* imagers have *high* visual excitability. For example, people with the sensory condition *synaesthesia* tend to score highly on mental imagery questionnaires (e.g., Barnett & Newell, 2008; Price, 2009; Spiller et al., 2015, but see Dance et al., 2021; Simner, 2013), while also showing high visual cortex excitability compared to controls (Terhune, Murray, et al., 2015; Terhune, Song, et al., 2015; Terhune et al., 2011). Important to our purposes here, cortical excitability has also been implicated in other imagery domains too (e.g., engaging in motor imagery reduces motor thresholds; Fadiga et al., 1999; Hashimoto & Rothwell, 1999; Stinear et al., 2006). We propose, therefore, that people with aphantasia may have lower excitability in the visual cortex, and that this reduced excitability may extend to other sensory regions. If so, people with aphantasia may typically have poor imagery in more than one sense.

The role of cortical excitability in imagery also raises a second important question. Visual cortex excitability has also been linked to a second phenomenon, *sensory sensitivity* (e.g., Green et al., 2013; Wilkins et al., 1984). Sensory sensitivity is a disturbance in the way individuals react to incoming sensory stimuli from the outside world. For example, someone who is visually hyper-sensitive might find lights too glaring, and avoid bright environments (e.g., supermarkets). They might also experience greater discomfort and visual distortions (e.g., shimmering, and flashes) in the *Pattern Glare Ta*sk, in which participants are shown gratings (parallel lines) at particular spatial frequencies that trigger visual sensitivities (Braithwaite, Broglia, Bagshaw, et al., 2013; Evans & Stevenson, 2008; Ward et al., 2017; Wilkins et al., 1984). Visual discomfort and distortions themselves are a form of ‘visual stress’ (‘*pattern glare*’), indicative of visual sensitivity (Braithwaite, Broglia, Bagshaw, et al., 2013; Braithwaite, Broglia, Brincat, et al., 2013; Ward et al., 2017; Wilkins et al., 1984). Important to our purposes here is that sensory sensitivities – like imagery – appear to relate to cortical excitability (e.g., Green et al., 2013; Wilkins et al., 1984). Hyper-excitability in the cortex (i.e., lower phosphene thresholds; e.g., Stewart et al., 2001) has been found in sensory-sensitive populations (e.g., people with migraine) and is associated with heightened pattern glare in these individuals (Aurora et al., 1999, 2003; Aurora & Wilkinson, 2007; Brigo et al., 2012; Chadaide et al., 2007; Coutts et al., 2012; Huang et al., 2003, 2011; Mulleners et al., 2001; Wilkins et al., 1984; Young et al., 2004). Other research, too, shows that special populations who tend to be high in imagery (synaesthetes, noted above for having high visual cortex excitability; Terhune et al., 2011, 2015) report high levels of sensory sensitivity (Van Leeuwen et al., 2019; Ward et al., 2018, 2017), and increased susceptibility to pattern glare (Ward et al., 2017). Taken together, this research raises the possibility that excitation within the visual cortex may give rise to both imagery differences and sensitivity differences within the same individuals. Here, we draw an explicit link between these findings, hypothesising for the first time that imagery and sensitivity are linked. Specifically, we predict that individuals with *low/absent* imagery (aphantasics) might report lower sensory sensitivity than the average person.

So far, our predictions have linked imagery and sensory sensitivity, but we have implicitly focussed on *hyper*-sensitivity (e.g., high/low excitability in high/low imagery linked to high/low *hyper*-sensitivity). But properly speaking, sensory sensitivity encompasses both hyper- and hypo- sensitivities (Robertson & Simmons, 2013). A person who is hyper-sensitive (in the visual domain for example) may find bright lights too glaring and seek to avoid them, while a person who is hypo-sensitive may experience low responsivity (‘sensory dampening’) and actively *seek* visual stimulation (e.g., flick their fingers in front of the eyes; Ben-Sasson et al., 2009; Bogdashina, 2003; Simmons et al., 2009). Paradoxically, hyper- and hypo-sensitivities are often found within the same individual, either across sense modalities (e.g., avoiding bright lights, but seeking odours) or within modalities (e.g., disliking loud noises, but playing the same song over and over; Robertson & Simmons, 2013; Smees et al., 2020; Ward et al., 2017). Above, we hypothesised a link between imagery and hyper-sensitivity, but are there links to both hyper- *and* hypo-sensitivity? In fact, we predict lower sensory sensitivities in aphantasics in both hyper- and hypo-domains, because these domains have themselves been linked through mechanisms of *adaptation* (Takarae & Sweeney, 2017; Ward, 2019).

Adaptation is when we stop noticing the smell of someone's perfume, for example, after having been in their company for a while (Dalton, 2000; Takarae & Sweeney, 2017; Ward, 2019). Normal adaptation is driven by a reduction in neural activity in response to continuous or repeated sensory input (Takarae & Sweeney, 2017; Thompson & Spencer, 1966). Failures in adaptation are linked to high levels of cortical excitation and therefore hyper-sensory sensitivity (Green et al., 2015). Specifically, high cortical excitation may prevent neural adaptation, meaning a stimulus remains ‘prominent’ in attention and becomes overbearing (Takarae & Sweeney, 2017; Ward, 2019). However, this same failure in adaptation may also give rise to hypo-sensitivity through the prioritising of old stimuli (i.e., the stimuli we are not adapting to). Prioritising old stimuli means that new stimuli are not easily recognised, leading to sensory dampening (i.e., hypo-sensitivity; Takarae and Sweeney, 2017). This fact may explain why individuals who report high levels of hyper-sensitivity often report high levels of hypo-sensitivity (Robertson & Simmons, 2013; Ward et al., 2017), and taken together, all these facts also make predictions about people with aphantasia. If strong imagery is linked to heightened cortical excitability, and heightened cortical excitability is linked to hyper-sensitivity (but also hypo-sensitivity via problems with sensory adaptation), we would expect people with very *low* imagery (aphantasia) to have *lower* levels of hyper-/hypo-sensitivity, when compared to imaging controls.

We summarise our hypothesised link between imagery, sensory sensitivity, and cortical excitation in Figure 1. Our model suggests that aphantasia may be characterised by lower levels of visual cortex hyperexcitability, with this mediating both low imagery *and* low sensitivities to incoming sensory information. Our model makes several predictions. First, people with aphantasia (impoverished visual imagery) may report fewer sensory sensitivities within the visual sense, relative to imaging controls (Experiment 1). Second, we predict that people with aphantasia may in fact have poor imagery across *multiple* domains (visual, gustatory, olfactory etc; Experiment 1). If so, we predict, thirdly, that people with aphantasia may also therefore self-report lower *sensory sensitivities* across multiple domains (Experiment 1). Thirdly, we predict that the sensory sensitivities reported in Experiment 1 by people with aphantasia should be corroborated by behavioural findings in a subsequent study (Experiment 3): aphantasics should experience fewer visual distortions and less visual discomfort than controls in response to irritable visual gratings in a *Pattern Glare Task*. Finally, if our model holds true in the population at large, then we predict visual imagery and sensory sensitivity to be positively correlated in a general population sample (Experiment 2). In summary, by investigating, for the first time, the relationship between imagery and sensory sensitivity, the present research aims to enhance our understanding of imagery, sensory sensitivity, cortical excitation, and aphantasia.

**Figure 1.**
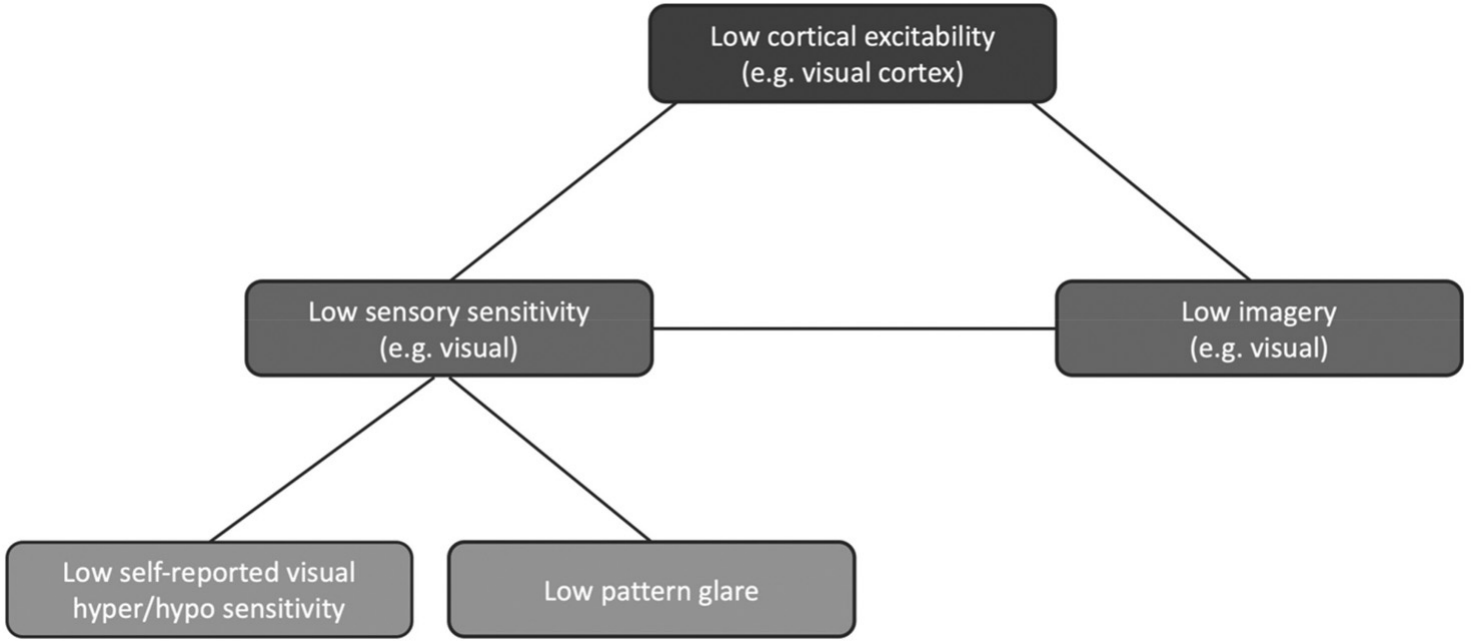
Excitability, Imagery, and its Measurements. Our proposed model links imagery, cortical excitation, and sensory sensitivity. Our model posits that low cortical excitability may be tied to low imagery (e.g., in people with aphantasia) and lower levels of sensory sensitivity. Our model is ambivalent about directionality (i.e., causality) and may be multi-directional. Indeed, levels of imagery (a ‘top-down’ process) and incoming sensory signals (a ‘bottom-up’ process) may influence levels of cortical excitability (or vice versa) on a moment-to-moment basis. We have presented our model in terms of *low* excitability to capture the experiences of aphantasics but note that the links proposed are correlational (i.e., it also predicts a link between *high* excitability/ imagery/ sensitivity). Here we have applied our model to *visual* imagery, but suggest it extends to the other sense domains also.

## Experiment 1

In this experiment we compared the profile of mental imagery and sensory sensitivity in people with and without aphantasia. We asked whether the imagery deficit reported by people with aphantasia extends across multiple sense domains, and whether this maps on to fewer sensory sensitivities. In our study we were mindful to factor out unwanted influences. Sensory sensitivities are not only variable within the general population (Horder et al., 2014; Robertson & Simmons, 2013) but are particularly characteristic of individuals with autism spectrum conditions (henceforth autism) (American Psychiatric Association, 2013) or non-clinical autistic traits (Ben-Sasson et al., 2009; Bogdashina, 2003; Horder et al., 2014; Robertson & Simmons, 2013, 2015; Simmons et al., 2009). Importantly, higher autistic traits are also found in people with aphantasia (Dance et al., 2021; Milton et al., 2021). These facts could potentially link imagery, aphantasia, and sensory sensitivities via the medium of autism. Participants in Experiment 1 were therefore screened using the *Autism Spectrum Quotient* (AQ; Baron-Cohen et al., 2001) and AQ scores were added into our analyses of sensory sensitivity as covariates. Note, however, that our predictions stand in the opposite direction to any confound. If people with aphantasia had any predisposition with regards to sensory sensitivities, their higher traits of autism would make this *more* likely. Instead, we are predicting *fewer* sensitivities for people with aphantasia, as an outcome of our model above (Figure 1). Our General Discussion explores this opposition more fully.

### Method

#### Participants

We recruited 164 aphantasics (101 female, 62 male, 1 other; *M* age = 42.35, *SD* = 15.95) and 138 controls (67 female, 70 male, 1 other; *M* age = 37.39, *SD* = 13.83)^1^. The majority of our aphantasic participants (*n* = 158) were recruited from the University of Sussex's *Imagery Lab - Aphantasia Cohort* while the remaining aphantasic participants (*n* = 6) were recruited from the student body of the University of Sussex. Control participants were recruited from multiple sources including Amazon's Mechanical Turk (MTurk; a platform for collecting quality data; Casler et al., 2013), and via word-of-mouth and social media.

Participants were separated into their two groups using the ‘gold standard’ questionnaire for aphantasia (*Vividness of Visual Imagery Questionnaire*; *VVIQ*; Marks, 1973, 1995) whose scores range from 16–80 (see below) and where a score between 16–32 is indicative of aphantasia (i.e., imagery is either absent or vague/dim; Keogh & Pearson, 2018; Zeman et al., 2015). The protocol for the VVIQ is given below, but we point out here that people show good metacognition about their own imagery abilities, and self-report measures such as this correlate well with behavioural validations (e.g., Keogh & Pearson, 2018; Pearson et al., 2011). Our aphantasics scored the required 16–32 on the VVIQ (*M* = 17.49, *SD* = 3.44), while our non-aphantasic controls scored above 32 (*M* = 59.67, *SD* = 11.93). As compensation for taking part, non-students were entered into a prize draw for a shopping voucher, and MTurk participants were given a monetary payment of $4 (for our 25 min test). For all experiments reported in this paper, participants provided informed consent prior to taking part, and ethical approval came from the *University of Sussex Sciences and Technology Cross-Schools Research Ethics Board*.

#### Materials and Procedure

Participants completed eight questionnaires online in a random order. (Our *Imagery Lab - Aphantasia Cohort* had already completed the VVIQ, so completed the remaining seven questionnaires.) Six questionnaires measured imagery (summarised in Table 1), while the final two questionnaires measured sensory sensitivities, and autistic traits. These eight measures are described below. Our study was hosted on our in-house testing platform (www.syntoolkit.org).

**Table 1.** Names and Domains of Imagery Questionnaires, with Abbreviations (Abbr.). Citations for these scales are given in the text below.

Domain	Questionnaire	Abbr.
Visual	Vividness of visual imagery questionnaire	VVIQ
	Plymouth sensory imagery questionnaire	Psi-Q
Auditory	Clarity of auditory imagery scale	CAIS
	Plymouth sensory imagery questionnaire	Psi-Q
Olfactory	Vividness of olfactory imagery questionnaire	VOIQ
	Plymouth sensory imagery questionnaire	Psi-Q
Tactile	Adapted shortened Betts’ questionnaire upon mental imagery Plymouth sensory imagery questionnaire	Betts-ad Psi-Q
Gustatory		
Bodily		
Movement	Vividness of movement imagery questionnaire kinaesthetic scale	VMIQ
Feeling	Plymouth sensory imagery questionnaire	Psi-Q

*Vividness of Visual Imagery Questionnaire (VVIQ;**Marks, 1973**).* The VVIQ was our aphantasia diagnostic. In this questionnaire, participants were asked to imagine a series of four scenarios (e.g., “A beach by the ocean on a warm summer's day”) and to rate the strength of their visual imagery for four aspects of each scene (e.g., “The appearance and colour of the water”). Imagery was rated on a five-point scale, comprising (1) (“No image at all, you only “know” that you are thinking of the object “), (2) (“Vague and dim”), (3) (“Moderately clear and vivid”), (4) (“Clear and reasonably vivid”), and (5) (“Perfectly clear and as vivid as normal vision”). Responses were summed to give scores ranging from 16–80. As noted above, responses between 16–32 (no imagery or vague/dim) are indicative of aphantasia.

*Clarity of Auditory Imagery Scale (CAIS;**Willander and Baraldi, 2010**).* Here, participants were asked to imagine a series of 16 sounds (e.g., “A dog barking”) and to rate how clearly they could ‘hear’ the sounds on a scale of 1 (“No sound at all, you only “know” that you are thinking of the sound”) to 5 (“Perfectly realistic and as vivid as the actual sound”). All scale-points here (and in the other imagery questionnaires with the exception of the Psi-Q; see below) mirror the wording of the VVIQ (for this wording, see above). To achieve this, we slightly re-worded the original response-scale (which had been: 1-‘Not at all’, to 5-‘Very clear’) to ensure consistency with other imagery questionnaires in our study (e.g., VVIQ). Total scores range from 16–80.

*Vividness of Olfactory Imagery Questionnaire (VOIQ;**Gilbert et al., 1997**).* Participants were asked to imagine four different odorous situations (e.g., “An outdoor cookout or barbeque”). Participants rated the vividness of their olfactory imagery for four aspects of each scenario (e.g., “The charcoal or wood has just been lit and is beginning to burn”) on a scale from 1 (“No odour at all, you only “know” that you are thinking of the odour”) to 5 (“Perfectly realistic and as vivid as the actual odour”). Total scores range from 16–80.

*Adapted Shortened Betts’ Questionnaire Upon Mental Imagery (**Sheehan, 1967**;**Spiller et al., 2015**).* We used this questionnaire to measure imagery for taste, tactile, and bodily sensations. Participants were asked to imagine tasting 12 items (e.g., “Coffee”), touching 12 items (e.g., “Sand”), and the experience of 12 bodily sensations (e.g., “Hunger”). Given the methodological issues noted in our Introduction, we used the version of the Betts’ questionnaire updated by Spiller et al. (2015). This version differs from the original (used by Dawes et al., 2020) in a number of ways. First, our version replaces out-dated items (e.g., fur muff → fur), and makes items sensorily clearer where necessary (e.g., jelly → strawberry jelly). Also, in place of just 5 items in the original, it uses 12 items per sense - and the novel items (n7 per modality) are again contemporary (e.g., “Clingfilm (plastic wrap, Saran wrap)”). Since Spiller et al.'s updates had been made to only three scales (taste, tactile, and bodily sensations), we used just these three scales in our own work. Participants rated their imagery on a scale from 1 (“No tactile sensation/taste/bodily sensation at all, you only “know” that you are thinking of the tactile sensation/taste/bodily sensation”) to 5 (“Perfectly realistic and as vivid as the actual tactile sensation/taste/bodily sensation”). Responses for each imagery scale (taste, tactile, bodily sensations) were summed separately, with possible scores ranging 12–60.

*Vividness of Movement Imagery Questionnaire 2 (VMIQ-2;**Roberts et al., 2008**).* Using the kinaesthetic subscale of this questionnaire, participants were asked to imagine the feeling of performing 12 movements (e.g., “jumping sideways”). The direction of the scoring scale was reversed to match our other imagery questionnaires, meaning participants rated vividness from 1 (“No image at all, you only “know” that you are thinking of the skill”) to 5 (“Perfectly clear and vivid as normal feel of movement”). Responses were summed, with possible scores ranging from 12–60.

*Plymouth Sensory Imagery Questionnaire (Psi-Q;**Andrade et al., 2014**).* This imagery questionnaire covers multiple domains, and was used in addition to our separate measures to ensure reliability of our results. Participants were asked to form a mental image of five items for each of the seven domains: visual (e.g., “a cat climbing up a tree”), auditory (e.g., “an ambulance siren”), olfactory (e.g., “a rose”), gustatory (e.g., “toothpaste”), tactile (e.g., “a soft towel”), bodily sensation (e.g., “having a sore throat”), feeling (e.g., “excited”). Participants rated each item on a scale from 0 (“No image at all”) to 10 (“Image as clear and vivid as real life”). This questionnaire provided imagery scores for each sense domain by averaging the items for each modality separately, with possible scores 0–10.

*Glasgow Sensory Questionnaire (GSQ;**Robertson & Simmons, 2013**).* This 42 item questionnaire measures sensory sensitivity across seven sense domains (visual, auditory, olfactory, tactile, gustatory, vestibular, and proprioceptive) with six items per sense. Within each sense, half of the items (*n* = 3) measure hyper-sensitivity and half measure hypo-sensitivity. Examples for hyper-sensitivity include “Do bright lights ever hurt your eyes/cause a headache?” (visual) and items for hypo- sensitivity include “Do you really like listening to certain sounds (for example, the sound of paper rustling)?” (auditory). Each question is rated on a scale of 0 (“Never”), 1 (“Rarely”), 2 (“Sometimes”), 3 (“Often”), and 4 (“Always”). The measure outputs an overall sensitivity score summed across all items (ranging 0 to 168), as well as one score for each of the seven senses (e.g., auditory; ranging 0 to 24). It also produces two scores collapsed over senses for hypo- and hyper-sensitivity respectively (ranging from 0 to 84 each).

*The Autism Spectrum Quotient (**Baron-Cohen et al., 2001**).* This 50 item questionnaire asks about five types of autism symptomatology: imagination (e.g. “I find making up stories easy”; reversed scored), social skills (e.g. “I find it hard to make new friends”), communication (e.g. “I frequently find that I don't know how to keep a conversation going”), attention switching (e.g. “I prefer to do things the same way over and over again”), and attention-to-detail (e.g. “I tend to notice details that others do not”). Participants rated their agreement on a 4-point scale: “Definitely agree”, “Slightly agree”, “Slightly disagree”, “Definitely disagree”. Responses are scored 1 for slightly/definitely agreeing with autism traits (poor imagination, communication, social skills, attention switching, but good attention-to-detail), or 0 otherwise. Scores of ≥32 are taken as the usual suggestive threshold for autism (Baron-Cohen et al., 2001). However, following Dance et al. (2021), we excluded one item that directly taps aphantasia phenomenology (‘I find it very easy to create a picture in my mind’), leaving AQ scores ranging from 0 to 49.

### Results

#### Do Aphantasics Have Poor Imagery in Multiple Domains?

**Figure 2.**
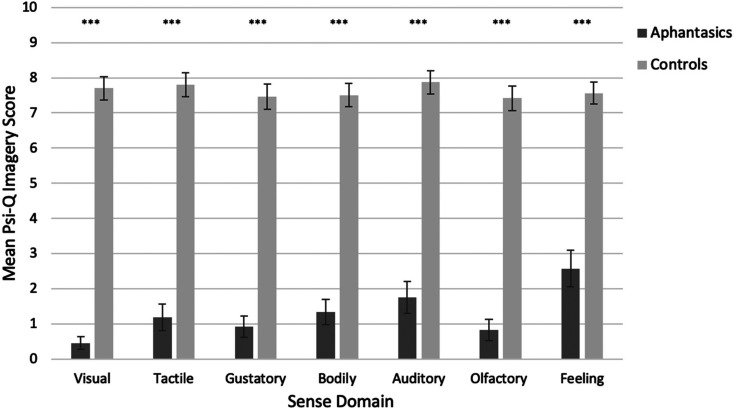
Mean imagery scores (with 95% confidence intervals) as a function of group (aphantasia, control) and sense domain using the Psi-Q. Higher scores indicate stronger imagery (scores for each sense domain are on the same scale ranging from 0-10).

*Note.* *** uncorrected *p* < .001.

Using the Psi-Q (i.e., our measure of imagery across multiple sense domains), we first conducted a 2 × 7 ANOVA crossing group (aphantasia, controls) with sense (visual, auditory, tactile, olfactory, gustatory, bodily, feeling). Aphantasics reported significantly weaker imagery than controls overall (*F*(1, 300) = 858.17, *p* < .001, $\eta_{p}^{2}=.741$; aphantasics: *M* = 1.29; *SD* = 1.94; controls: *M* = 7.62; *SD* = 1.78) and there was also a main effect of sense domain (*F*(3.91, 1,173.01) = 22.72, *p* < .001, $\eta_{p}^{2}=.070$; with Greenhouse-Geisser correction; e.g., olfaction was a weaker imagery domain, see Figure 2). There was also a significant interaction (*F*(3.91, 1,173.01) = 20.17, *p* < .001, $\eta_{p}^{2}=.063$; with Greenhouse-Geisser correction) because group differences were more pronounced for some senses over others (e.g., more pronounced for vision, less for feeling; see Figure 2). However, group differences were significant (i.e., aphantasics had weaker imagery) for every sense modality, using a series of bootstrapped independent samples t-tests with Welch correction (all *p* < .001, with large hedges’ *g* effect sizes = 1.77–4.48; see Table S1 in our Supplementary Information, SI, for further details). Here, and throughout our manuscript, we report bias-corrected and accelerated (BCa) bootstrapped confidence intervals (with bootstrapping performed 1,000 times) for group comparisons where deviations from normality are present in our data. After vision, tactile imagery was most affected, followed by olfactory, gustatory, bodily, auditory, and feeling imagery (see Figure 2). All differences survive correction for the multiple pairwise comparisons performed using the Benjamini-Hochberg False Discovery Rate method (Benjamini & Hochberg, 1995, 2000).

**Figure 3.**
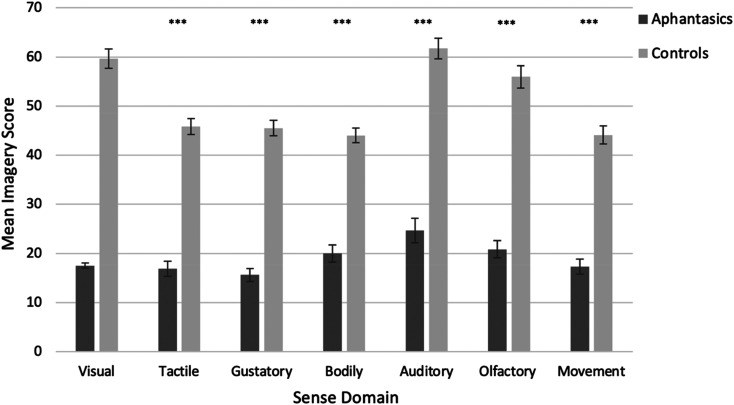
Mean imagery scores (with 95% confidence intervals) as a function of group (aphantasia, control) and sense domain using the independent sensory imagery questionnaires (visual; *VVIQ*, auditory; *CAIS*, olfactory; *VOIQ*, gustatory; *Betts-ad*, tactile; *Betts-ad*, body sensation; *Betts-ad*, movement; *VMIQ*). Higher scores indicate stronger imagery (maximum possible scores for each sense domain vary depending on the questionnaire; see methods).

*Note.* *** uncorrected *p* < .001 (we did not conduct a group comparison of VVIQ scores because we used this measure to categorise people as aphantasic; see methods).

Next, we sought to replicate our findings using our other sensory imagery questionnaires (auditory *CAIS*; olfactory *VOIQ*; gustatory *Betts-ad*; tactile *Betts-ad*; body sensation *Betts-ad*; movement *VMIQ*). Given that our imagery questionnaires were independent measures and had different total possible scores (see methods), we ran a series of bootstrapped independent samples t-tests with Welch correction comparing aphantasics to controls. Again, group differences were significant for every sense (i.e., aphantasics had weaker imagery; all *p* < .001, with large hedges’ *g* effect sizes 2.33–3.37; see Table S1 in our SI for further details). These differences are depicted visually in Figure 3, and all differences again survive correction for multiple pairwise comparisons.

Next, we asked whether the imagery deficits seen in our aphantasics were severe enough to be considered ‘aphantasia-like’ (i.e., imagery is entirely absent or vague/dim), even outside the visual domain. We therefore looked within each independent sensory imagery questionnaire (visual; *VVIQ*, auditory; *CAIS*, olfactory; *VOIQ*, gustatory; *Betts-ad*, tactile; *Betts-ad*, body sensation; *Betts-ad*, movement; *VMIQ*) and identified participants who scored within the standard aphantasia range (imagery is absent or vague/dim: scoring 16–32 on the *VVIQ*, *CAIS*, *VOIQ*; and 12–24 on *Betts-ad*, *VMIQ*), albeit for non-visual imagery. We found that 159 (97%) aphantasics showed aphantasia-like imagery weakness in at least one other (non-visual) domain, compared to 15 controls (11%), which was highly significant (*χ*^2^ (1, *N* = 302) = 223.89, *p* < .001; using Yates correction). Moreover, 101 aphantasic individuals (62%) had aphantasia-like imagery (absent or vague/dim) across *all* other sense domains, compared to 0 controls (*χ*^2^ (1, *N* = 302) = 124.94, *p* < .001), and 56 aphantasics (34%) reported *no imagery at all* (not vague/dim) in *any domain at all* (compared to 0 controls; *χ*^2^ (1, *N* = 302) = 55.61, *p* < .001). In summary, aphantasics not only had weaknesses in *visual* imagery, but almost always (97%) had another imagery deficit (e.g., olfactory), and sometimes (62%) had deficits across *all* imagery modalities.

#### Does Imagery Predict Sensory Sensitivity?

Next, we asked whether aphantasics reported fewer sensory sensitivities than controls by looking at the GSQ. We conducted a 2 × 2 × 7 ANCOVA crossing group (aphantasic, control) with sensitivity type (hyper-, hypo-sensitivity) and sense domain (GSQ; visual, auditory, olfactory, tactile, proprioception, vestibular, gustatory). We included AQ scores as a covariate to control for the influence of autism traits. The ANCOVA revealed a significant main effect of group (*F*(1, 299) = 27.08, *p* < .001, $\eta_{p}^{2}=.083$, with aphantasics reporting fewer sensitivities overall (*M* = 50.52, *SD* = 22.88) relative to controls (*M* = 63.62, *SD* = 32.89).

**Figure 4.**
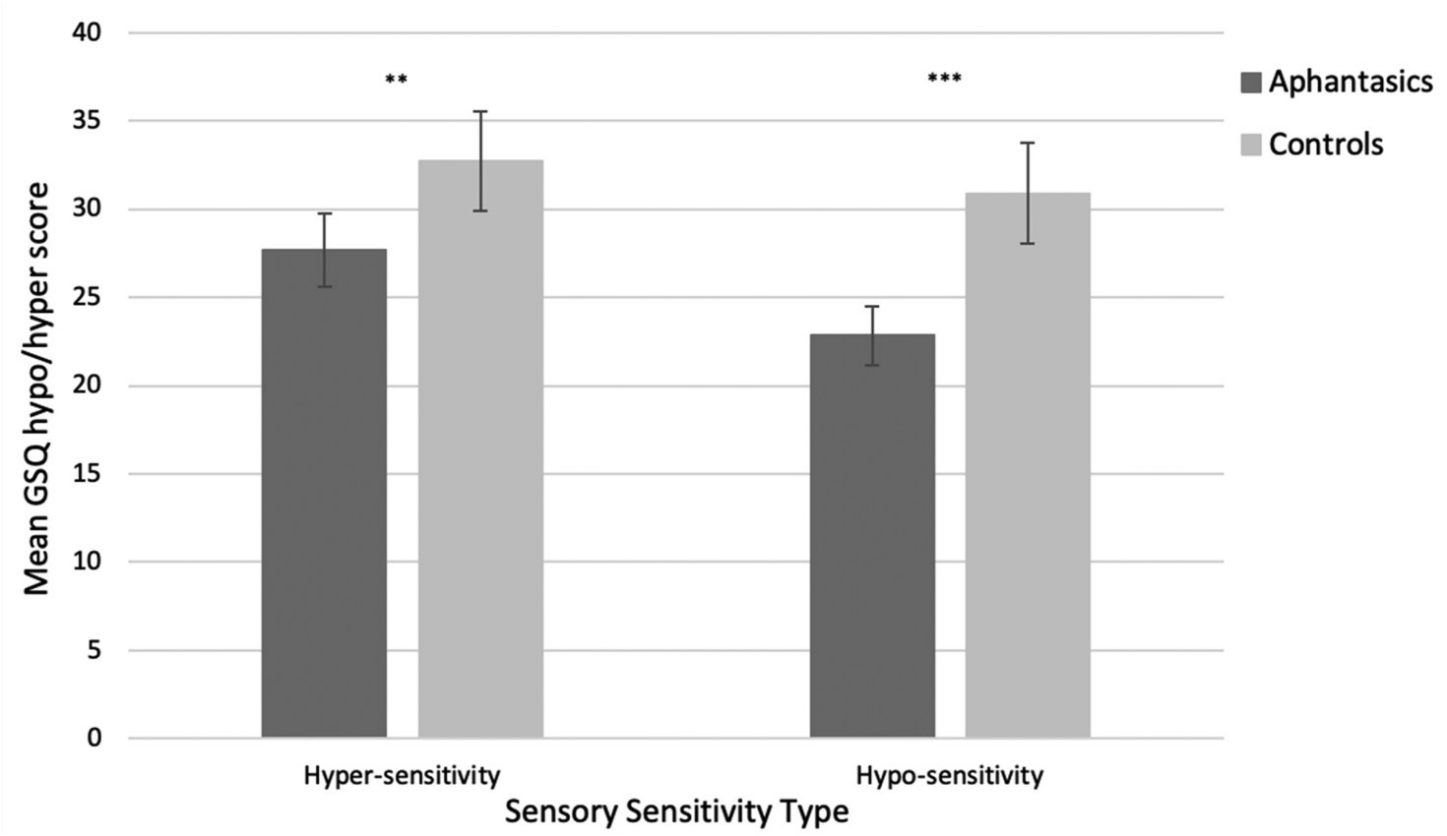
Mean hypo- and hyper-sensitivity scores (with 95% confidence intervals) as a function of group (aphantasia, control) using the GSQ. Higher scores indicate higher levels of sensory sensitivity.

*Note.* ** uncorrected *p* < .01, *** *p* < .001.

Although there was no significant main effect of sensitivity type (hyper/hypo), (*F*(1, 299) = 1.81, *p* = .277, $\eta_{p}^{2}=.004$; with Greenhouse-Geisser correction), there was a significant interaction between sensitivity-type and group (*F*(1, 299) = 9.16, *p* = .003, $\eta_{p}^{2}=.030$; with Greenhouse-Geisser correction). Figure 4 suggests that although aphantasics are less sensitive than controls for both hyper-sensitivities and hypo-sensitivities, the effect is smaller for the former. We conducted two comparisons using bootstrapped independent samples t-tests with Welch correction to confirm that aphantasics reported significantly fewer hyper-sensitivities (*M* = 27.68; *SD* = 13.60), (*t*(263.81) = 2.84, *p* = .005, *g* = 0.33, BCa 95% CI [1.78, 8.37])), *and* hypo-sensitivities (*M* = 22.84, *SD* = 10.80), (*t*(224.09) = 4.81, *p* < .001, *g* = 0.58, BCa 95% CI [5.05, 11.17])), than controls (hyper: *M* = 32.72, *SD* = 16.68; hypo: *M* = 30.90, *SD* = 17.00; see Figure 4). Both differences survive correction for multiple pairwise comparisons using the Benjamini-Hochberg False Discovery Rate method (Benjamini & Hochberg, 1995, 2000).

**Figure 5.**
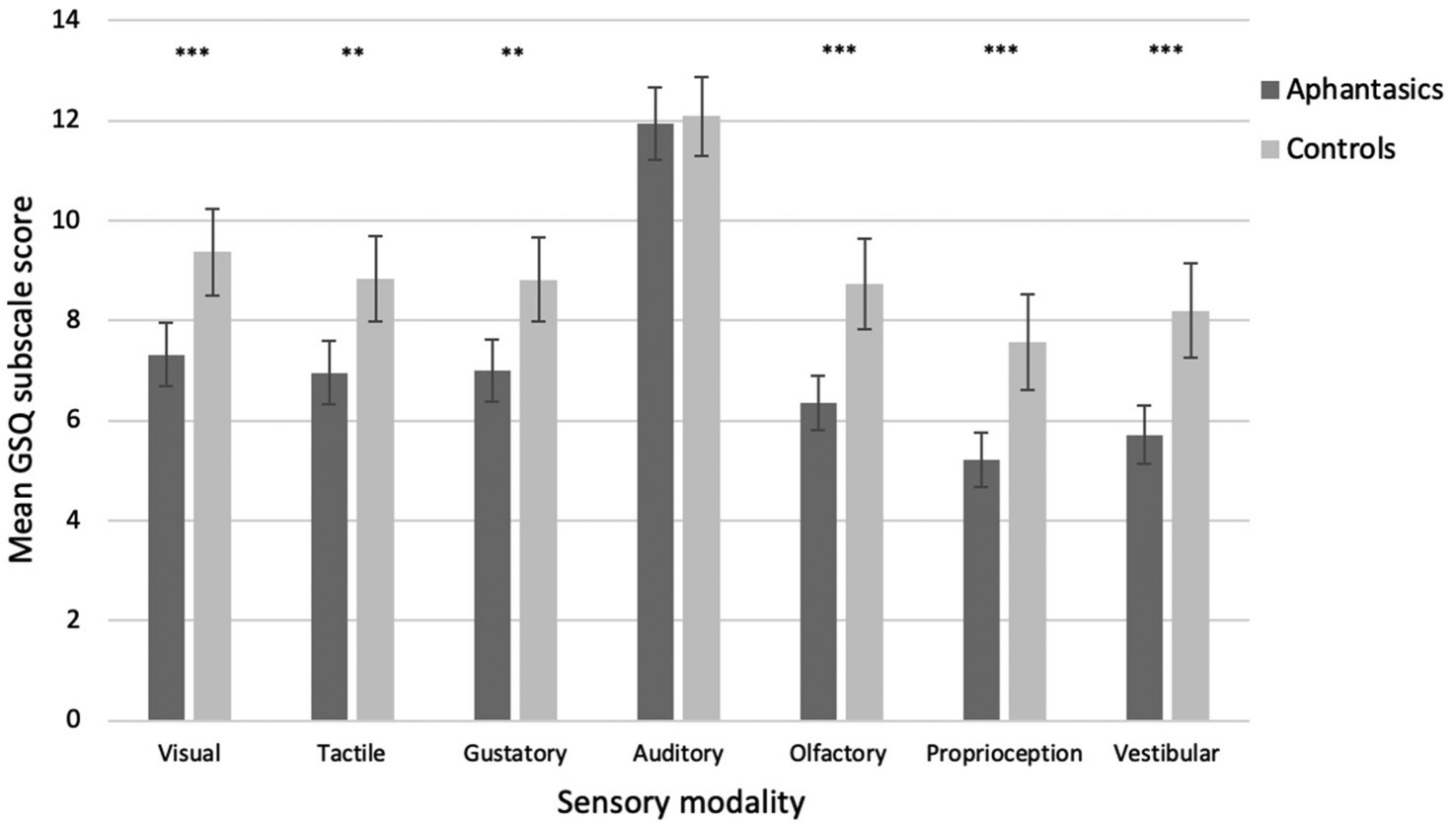
Mean overall sensory sensitivity scores (with 95% confidence intervals) as a function of group (aphantasia, control) and sense domain using the GSQ. Higher scores indicate higher levels of sensory sensitivity (encompassing hypo- and hyper-sensitivity).

*Note.* ** uncorrected *p* < .01, *** *p* < .001.

Our ANCOVA also revealed a significant interaction between group and sense domain, (*F*(5.14, 1,538.13) = 7.41, *p* < .001, $\eta_{p}^{2}=.024$; with Greenhouse-Geisser correction). To explore this interaction, we conducted a series of comparisons using bootstrapped independent samples t-tests with Welch correction, depicted visually in Figure 5 (see SI Table S2 for further information). Aphantasics reported significantly fewer sensitivities in the visual, olfactory, tactile, gustatory, vestibular, and proprioception domains, relative to controls (all *p* < .01, *g* = 0.41–0.54). All differences here survive correction for multiple comparisons. There was, however, no difference between aphantasics and controls in auditory sensory sensitivity (*p* = .791, *g* = 0.03). A Bayes Factor (using R version 3.5.1, R Core Team, 2018; Bayes Factor version 4.2, Morey and Rouder, 2018) of 0.13 suggested no group differences, with moderate confidence (assuming BF < .33 is taken as evidence for the null hypothesis; Dienes, 2014). Overall, this shows that with the exception of audition, aphantasics report lower levels of sensory sensitivity across all sensory modalities in comparison to imaging controls, while also controlling for the influence of autism traits.

Our ANCOVA also revealed other effects unrelated to our hypotheses (which we did not explore further, to reduce proliferation of multiple comparisons). For example, there was a significant main effect of AQ score, (*F*(1, 299) = 92.94, *p* < .001, $\eta_{p}^{2}=.237$) and sense domain, (*F*(5.14, 1,538.13) = 12.60, *p* < .001, $\eta_{p}^{2}=.040$; Greenhouse-Geisser correction). As expected from the autism literature (American Psychiatric Association, 2013) higher AQ scores linked with higher sensory sensitivity, and as expected from the sensitivity literature (Robertson & Simmons, 2013), some sense domains were more sensitive than others (e.g., auditory domain most sensitive; see Figure 5).

### Discussion

Here, we found that aphantasics experience significantly weaker imagery than controls across multiple sense domains, and are significantly more likely to have aphantasia-like imagery-weakness across multiple (and even all) senses compared to controls. Indeed, 97% of aphantasics (i.e., almost every one) had imagery deficits not only in the visual domain but also in at least one other. Aphantasics also reported fewer sensory sensitivities overall (in both hyper- and hypo-sensitivity), and fewer sensitivities within each of the senses with the exception of audition. In sum, our results show that imagery and sensory sensitivity are related: people with aphantasia experience lower levels of imagery and sensory sensitivity across multiple sense domains.

## Experiment 2

In Experiment 1 we demonstrated that imagery and sensory sensitivity are linked. But we are left with the question of whether the link between imagery and sensory sensitivity is seen specifically in aphantasic people, or whether it applies to the general population also. In Experiment 2 we addressed this question, by examining whether there is an association between visual imagery and sensory sensitivity in a student general population sample.

### Method

#### Participants

Our participants were 83 undergraduate students registered at the University of Sussex (63 females, 20 males; *M* age = 19.87, *SD* = 3.60). Participants took part in our study in return for research participation credits, and were sampled without mention of imagery or aphantasia in order to represent a random sampling of the population. Six of these participants had VVIQ scores in the aphantasia range (16–32), but they are included here because their scores represent part of the natural continuum of imagery within a general population sample.

#### Materials and Procedure

Participants completed the VVIQ (Marks, 1973), GSQ (sensory sensitivity measure; Robertson and Simmons, 2013), and the AQ (to factor out autistic traits; Baron-Cohen et al., 2001) in a random order. Details of these three measures are described in Experiment 1. Participants were provided with a URL to the testing site (www.syntoolkit.org) and they completed the study from their own homes.

### Results

**Figure 6.**
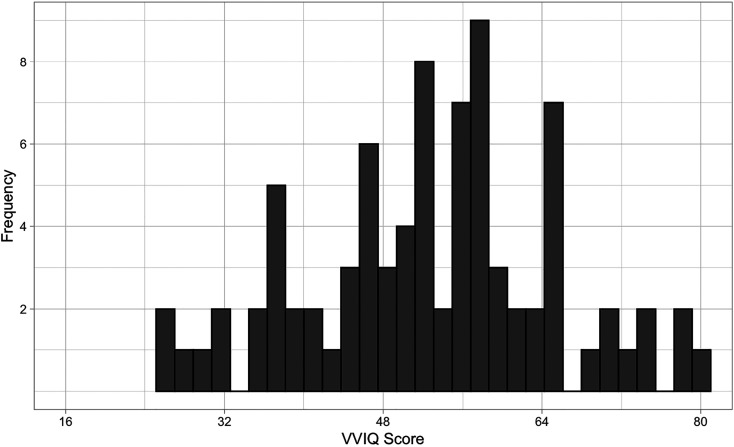
A histogram to show the distribution of VVIQ scores in our student general population sample.

To examine whether there was an association between imagery and sensory sensitivity we conducted a linear regression (using the enter method) predicting sensory sensitivity (GSQ) from visual imagery (VVIQ) and autism traits (AQ score). We constructed two regression models, the first predicting GSQ score from AQ score, and the second adding VVIQ score as an additional predictor. Our data was normally distributed, and the residuals in our models met the required assumptions for parametric tests (so confidence intervals were not bootstrapped). Both model one (*F*(1, 81) = 21.35, *p* < .001) and model two (*F*(2, 80) = 13.18, *p* < .001) significantly predicted sensory sensitivity scores. Model two was a significantly better model than model one, explaining 24.8% of the variance in sensory sensitivity scores (3.9% more than model one), (*F*(1, 80) = 4.17, *p* = .045). As expected from prior literature (Robertson & Simmons, 2013), model two revealed a significant positive relationship between autism traits (AQ) and sensory sensitivity (GSQ score; *b* = 1.24, SE(*b*) = .27, *t* = 4.61, *p* < .001, 95% CI [.706, 1.78]), showing that sensory sensitivity increased with levels of autism traits. Importantly, model two also showed a significant positive association between VVIQ score and GSQ score, (*b* = .26, SE(*b*) = .13, *t* = 2.04, *p* = .045, 95% CI [.007, .519]), indicating that as visual imagery scores increased, so did overall sensory sensitivity scores. Overall, our analysis shows that even when taking into account the influence of autistic traits (std. *b* = .45), visual imagery is a significant positive predictor (std. *b* = .20) of sensory sensitivity (see Figure 6 for the distribution of VVIQ scores).

### Discussion

In Experiment 2 we investigated the relationship between visual imagery and sensory sensitivity in a general population sample taken from a student body. We found that imagery and sensory sensitivity are positively correlated, even when autistic traits are factored out. Our results therefore show that imagery is not only associated with levels of sensory sensitivity in people with aphantasia (Experiment 1), but that this relationship is also evident in the general population (Experiment 2). In other words, while in Experiment 1 we found that people with aphantasia (who are low in imagery) tend to experience reduced levels of sensory sensitivity, here we found further support in the general population: as visual imagery level increases, so does overall sensory sensitivity.

## Experiment 3

In Experiments 1 and 2 we demonstrated that people who report lower visual imagery also report lower sensory sensitivities – both in aphantasia, and in the general population. Experiment 3 aims to validate our findings using behavioural evidence from a *Pattern Glare Task*. In response to gratings designed to elicit pattern glare, we asked whether people with poor imagery would experience less visual sensory sensitivity (less visual discomfort, and fewer visual distortions) than we might otherwise expect. Here, we compared a sample of people with aphantasia (low imagery) to controls with typical visual imagery abilities. We predict that the former will show lower pattern glare effects, indicative of lower sensory sensitivity.

### Method

#### Participants

Our participants were 56 aphantasics (28 female, 26 male, 2 other; *M* age = 33.66; *SD* = 8.36) and 56 controls (39 female, 17 male; *M* age = 29.84, *SD* = 16.91). Participants were matched in age (*t*(80.39) = −1.52, *p* = .133). Controls were recruited from the same sources as Experiment 1, with the addition of the undergraduate student body at the University of Sussex, and excluding Amazon's Mechanical Turk. Most of our aphantasic participants were recruited from the University of Sussex's *Imagery Lab - Aphantasia Cohort* (*n* = 54), with the remaining participants (*n* = 2) recruited via the same sources as controls. Participants were verified as aphantasics in the same way as in Experiment 1 (scoring 16–32 on the VVIQ; *M* = 17.77, *SD* = 3.86; compared to controls who scored >32 on the VVIQ; *M* = 58.89, *SD* = 10.62). Of our participants, 24 also took part in Experiment 1 (19 aphantasics, 5 controls), and 5 also took part in Experiment 2 (5 controls). Participants were advised not to take part in the study if they had a history of epilepsy given that uncomfortable visual patterns can trigger photosensitive epilepsy (Wilkins et al., 1980). Participants were compensated in the same way as Experiment 1.

#### Materials and Procedure

All participants completed the pattern glare task online using the testing platform Inquisit (*Inquisit 5 [Computer software], 2016*). After the pattern glare task, participants completed the VVIQ, with the exception of participants who had already completed it previously. Please see Experiment 1 for a description of these latter participants (from our *Imagery Lab - Aphantasia Cohort*), as well as the VVIQ protocol.

*Pattern Glare Task.* Participants viewed achromatic gratings, which were oval in shape and consisted of black and white horizontal stripes, presented on a grey background (RGB 128, 128, 128). Our version was based on Ward et al. (2017), who used gratings from Braithwaite, Broglia, Brincat, et al. (2013). There were three types of grating, each differing in spatial frequency: *low* (approx. 0.4 cycles per degree; cycles per degree is a measure of spatial frequency, see Braithwaite, Broglia, Bagshaw, et al., 2013; Ward et al., 2017; Wilkins et al., 1984), *medium* (approx. 3 cycles per degree), and *high* (approx. 10 cycles per degree). The low spatial frequency grating acted as a baseline stimulus, whilst the medium and high spatial frequency gratings were ‘irritable’ gratings expected to elicit ‘pattern glare’ (Braithwaite, Broglia, Bagshaw, et al., 2013; Braithwaite, Broglia, Brincat, et al., 2013; Conlon et al., 2001; Evans & Stevenson, 2008; Wilkins et al., 1984). Gratings were presented at their actual size of 230.01 mm wide × 176.39 mm high in the centre of the screen, and participants were instructed to sit 80 cm/32 inches from the monitor. A calibration procedure ensured that gratings were presented at the standardised size (approx. 12.58 degrees in height) on each computer monitor (participants adjusted the length of a line to match the length of a bank card, to calculate the number of pixels required for the gratings to reach the standardised size).

**Figure 7.**
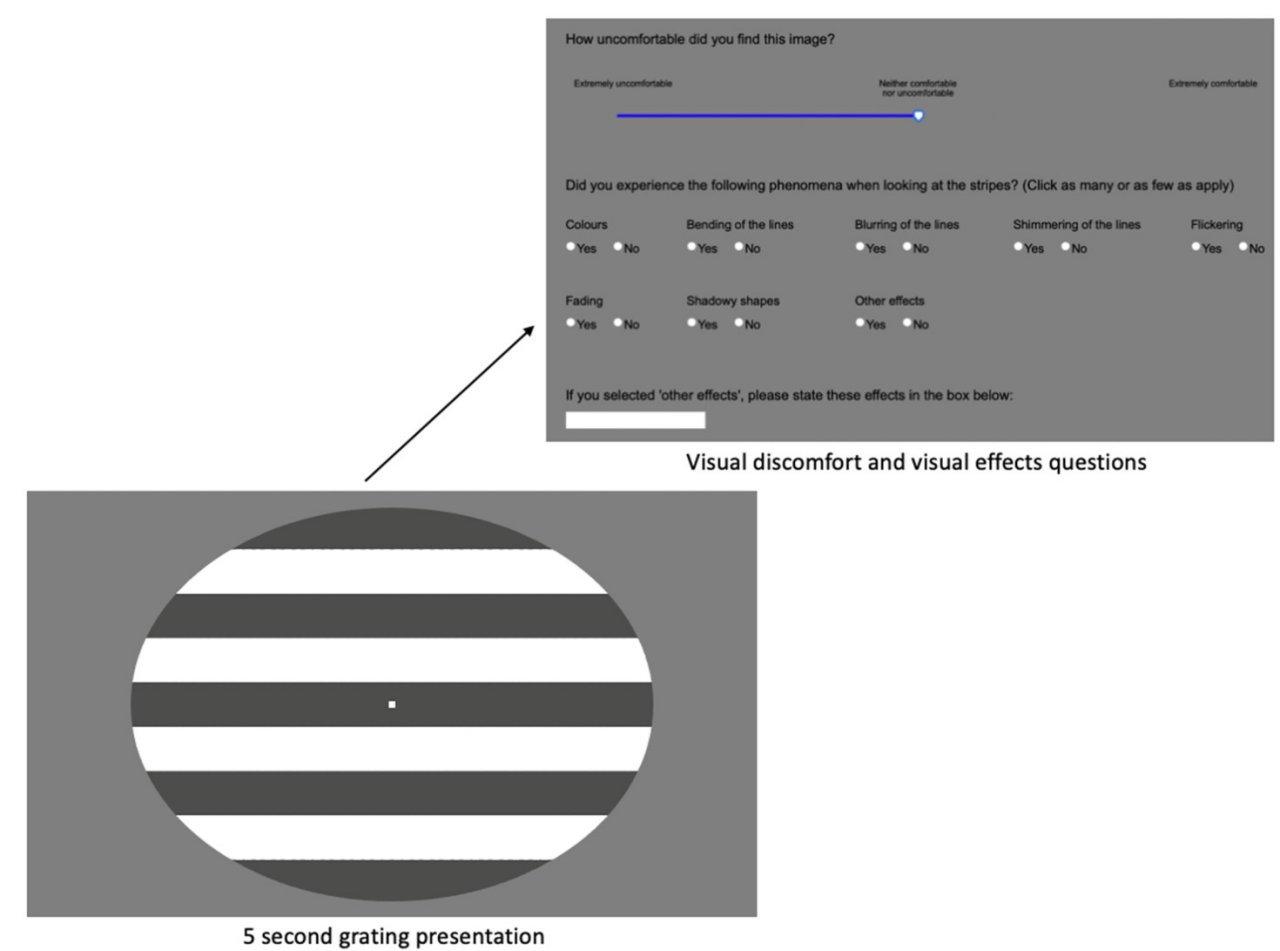
The trial sequence in the Pattern Glare Task (using an example of a low spatial frequency grating). In each trial the grating was presented for 5 s, followed by questions about visual discomfort and visual effects experienced.

Participants were told they should fixate their gaze on the centre of each pattern presented. Each grating was presented twice (each for 5 s) in a random order (6 trials overall). Following each grating (i.e., each trial), participants were asked two questions: how uncomfortable the image was, and how many visual effects were experienced. Comfort was measured on a sliding scale from 1 (“Extremely uncomfortable”) to 11 (“Extremely comfortable”), and visual effects were reported by checking any that applied (colours, bending of the lines, blurring of the lines, shimmering of the lines, flickering, fading, shadowy shapes, other effects; total effects for each grating ranging from 0–8). See Figure 7 for an example of the trial sequence. For each grating type (low, medium, high) the visual discomfort ratings and the number of visual effects elicited were averaged separately. Participants began the task with a practise grating of black and white checkers.

### Results

For each of our measures (see below), we conducted a 2 × 3 ANOVA crossing group (aphantasic, control) and grating spatial frequency (high, medium, and low). These were followed by bootstrapped independent samples t-tests with Welch correction to examine differences in response to the gratings designed to elicit visual sensory sensitivity (medium- and high-spatial frequency). We did not conduct pairwise comparisons for the low (baseline) grating to avoid proliferation of multiple comparisons, and because our hypotheses did not motivate this (i.e., it has been well established that low spatial frequency gratings do not trigger visual sensory sensitivity; e.g., Braithwaite, Broglia, Bagshaw, et al., 2013; Evans & Stevenson, 2008; Ward et al., 2017; Wilkins et al., 1984).

**Figure 8.**
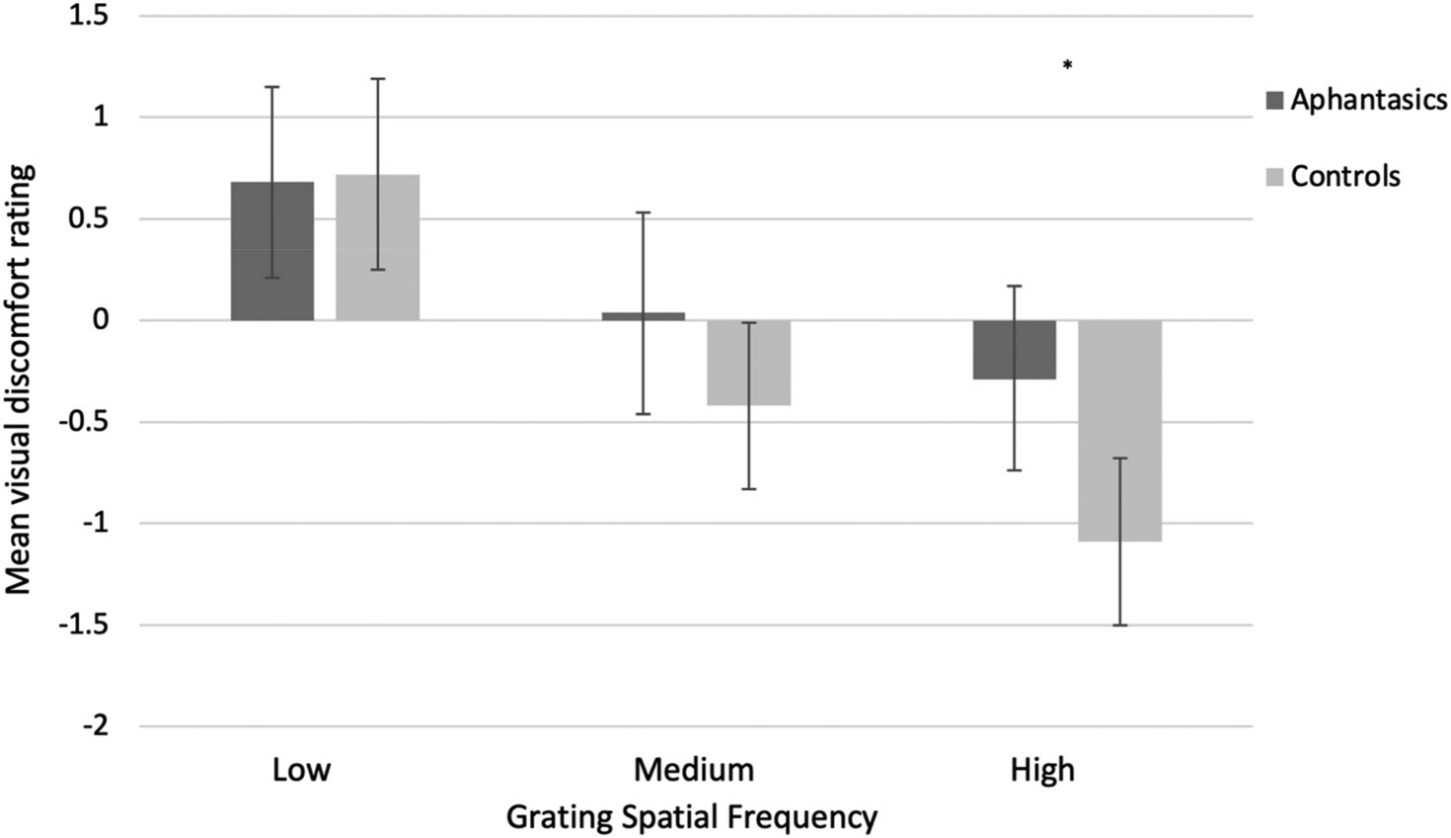
Mean visual discomfort ratings (with 95% confidence intervals) as a function of group (aphantasia, control) and grating spatial frequency (low, medium, high). For ease of visual display, scores were re-coded from a scale of 1–11 to a scale of −5 to 5 where scores above 0 indicate visual ‘comfort’, and scores below 0 indicate visual ‘discomfort’.

*Note.* * uncorrected *p* < .05.

The ANOVA for visual discomfort ratings revealed the expected main effect of spatial frequency (*F*(1.78, 195.81) = 46.25, *p* < .001, $\eta_{p}^{2}=.269$; with Greenhouse-Geisser correction), with more visual discomfort from the high (*M* = 5.31, *SD* = 1.67) and medium gratings overall (*M* = 5.81, *SD* = 1.71), relative to the low (baseline) grating (*M* = 6.70, *SD* = 1.75). There was no significant main effect of group (*F*(1, 110) = 2.21, *p* = .140, $\eta_{p}^{2}=.020$), but there was a significant interaction (*F*(1.78, 195.81) = 4.25, *p* = .019, $\eta_{p}^{2}=.037$; with Greenhouse-Geisser correction). Although there was no significant difference in discomfort for the medium grating between aphantasics (*M* = 6.04, *SD* = 1.84) and controls (*M* = 5.58, *SD* = 1.54; *t*(106.73) = −1.42, *p* = .159, *d* = 0.27, BCa 95% CI [−1.09, .156]; Bayes factor = 0.49, i.e., anecdotal support for null hypothesis), there was a significant difference for the high spatial frequency grating. Here, aphantasics reported significantly less visual discomfort (*M* = 5.71, *SD* = 1.70) than controls (*M* = 4.91, *SD* = 1.55), (*t*(109.03) = -2.62, *p* = .010, *d* = 0.49, BCa 95% CI [−1.39, −.249]). This demonstrates that although both groups found the high grating uncomfortable (scores <6 indicate visual ‘discomfort’, and scores >6 indicate visual ‘comfort’), aphantasic individuals reported less discomfort than controls (see Figure 8, where scores are recoded around zero for ease-of-display). This result survives when correcting for the multiple comparisons performed using the Benjamini-Hochberg False Discovery Rate method (Benjamini & Hochberg, 1995, 2000).

**Figure 9.**
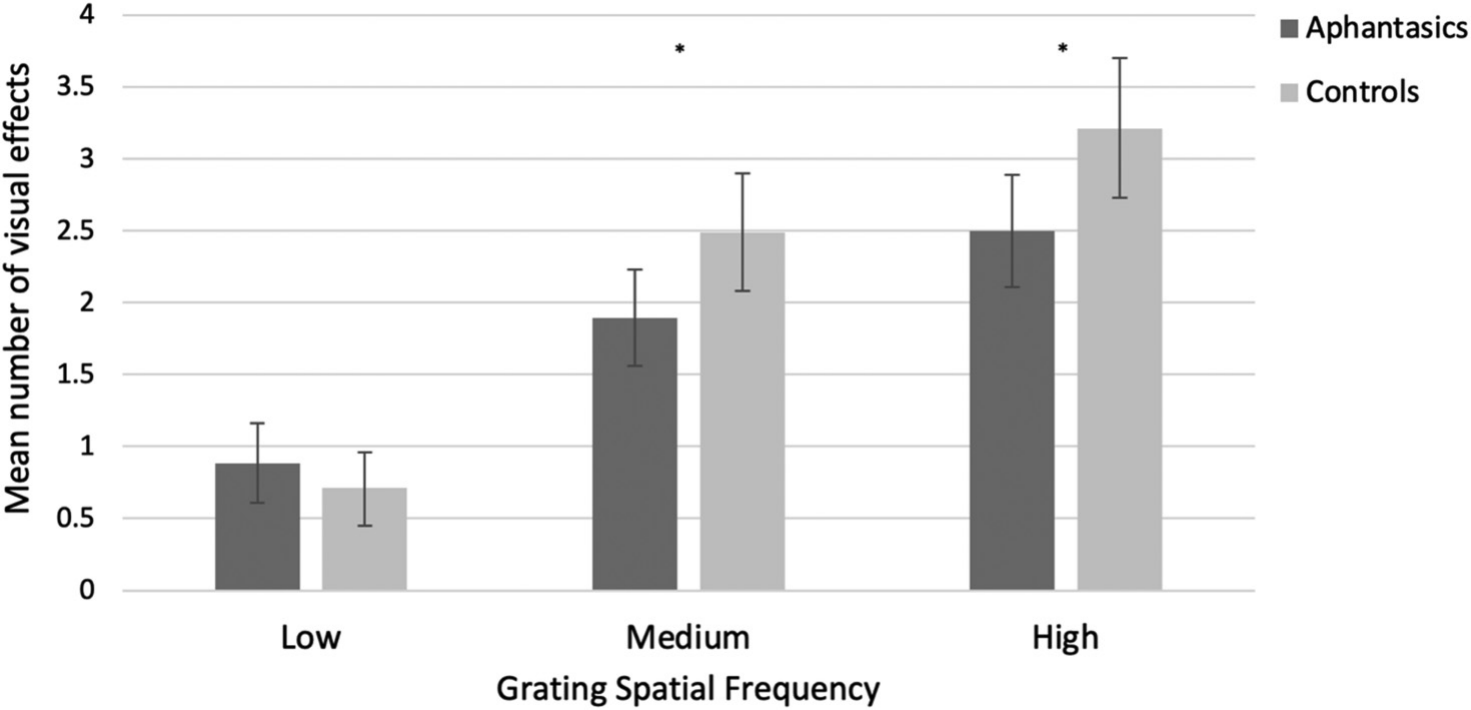
Mean number of visual effects (with 95% confidence intervals) as a function of group (aphantasia, control) and grating spatial frequency (low, medium, high).

*Note.* * uncorrected *p* < .05.

We next looked at the *number* of visual effects for each participant. As before, and as expected, there was a main effect of spatial frequency, (*F*(1.82, 199.77) = 130.70, *p* < .001, $\eta_{p}^{2}=.543$; with Greenhouse-Geisser correction) with more visual effects from high (*M* = 2.86, *SD* = 1.68) and medium gratings overall (*M* = 2.19, *SD* = 1.43), relative to the low (baseline) grating (*M* = .79, *SD* = 1.00). Our analysis also revealed a significant interaction (*F*(1.82, 199.77) = 6.95, *p* = .002, $\eta_{p}^{2}=.059$; with Greenhouse-Geisser correction) because group differences were more pronounced for some gratings than others (e.g., for the high grating; see below and Figure 9), but no significant effect of group, (*F*(1, 110) = 3.18, *p* = .077, $\eta_{p}^{2}=.028$).

As before, for the high spatial frequency grating, aphantasics reported significantly fewer visual effects (*M* = 2.50, *SD* = 1.44) than controls (*M* = 3.21, *SD* = 1.83), (*t*(104.41) = 2.30, *p* = .024, *d* = 0.43, BCa 95% CI [.161, 1.22]). However, aphantasics also reported significantly fewer visual effects (*M* = 1.89, *SD* = 1.26) than controls (*M* = 2.49, *SD* = 1.53) in response to the medium grating (*t*(106.03) = 2.26, *p* = .026, *d* = 0.43, BCa 95% CI [.110, 1.06]); see Figure 9), and all differences survive correction for multiple comparisons.

### Discussion

Here, we again present evidence that people with aphantasia experience less visual sensory sensitivity than people with visual imagery. As predicted, aphantasics reported significantly less pattern glare than controls: high/medium gratings gave them fewer visual effects, and the high gratings gave them less visual discomfort. These results provide behavioural corroboration for Experiment 1, showing that people with aphantasia experience lower levels of sensory sensitivity than imaging controls.

## General Discussion

Our intention was to characterise the sensory imagery deficits and sensory sensitivities experienced by people with aphantasia, and in doing so, examine how imagery and sensory sensitivity may be linked. Until now, aphantasia has been characterised by an absence of *visual* imagery (e.g., Milton et al., 2021; Zeman et al., 2015, 2016, 2020). But in Experiment 1 we found that aphantasic individuals report significantly weaker imagery compared to controls within all of the sense domains we tested (visual, olfactory, tactile, gustatory, bodily sensation, feeling, movement). When considering imagery in other senses in terms of the same criteria as aphantasia (i.e., imagery that is absent/dim/vague), we found that almost 100% of people with aphantasia had poor, aphantasia-like imagery in at least one other sense (i.e., 97% of aphantasics, but just 11% of controls), and as many as 62% of aphantasics had imagery-weaknesses in *all* of their senses, compared to 0% of controls. In the most extreme cases, one third of aphantasics (34%) reported *no imagery whatsoever* in *any sense whatsoever*.

Our results are in line with research showing that imagery strength is often positively correlated across sensory modalities (Andrade et al., 2014; Leclerc et al., 2019; Lima et al., 2015; White et al., 1974), and fits with recent studies using different methods. For example, Dawes et al. (2020) found that 26% of aphantasics reported a complete lack of imagery across all sense domains they tested in their own study (compared to our 34%). Their paper was an important step in understanding the broader imagery phenomenology of aphantasia, although their imagery questionnaire (*Sheehan's adapted version of Betts’ Questionnaire Upon Mental Imagery*; Sheehan, 1967) has been criticised for its small number of items and outdated language (e.g., Pecher et al., 2009; Spiller et al., 2015). Nonetheless, our results converged with their own, allowing our paper to stand as a validation and replication of their findings using multiple contemporary imagery questionnaires (see Experiment 1). Our results also fit with personal reports from aphantasic writers (Watkins, 2018) and with data from 2,000 aphantasics asked to self-diagnose imagery deficits, in which 54% reported imagery weaknesses across all sense modalities (Zeman et al., 2020) – compared to our own 62%. In sum, our results provide robust evidence that people with aphantasia tend to self-report imagery that is weak across multiple senses.

An important question that emerges from our findings is *why* aphantasics often experience multi-modal imagery deficits. We have suggested that understanding the neural mechanisms underlying mental imagery may shed light on this. Engaging in imagery is associated with activation in the relevant sensory cortex (e.g., visual imagery tied with visual cortex) and this has been shown for visual (Cattaneo et al., 2011; Cui et al., 2007; Daselaar et al., 2010; Dijkstra et al., 2017; Ganis et al., 2004; Sparing et al., 2002), auditory (Daselaar et al., 2010; Zvyagintsev et al., 2013), olfactory (Djordjevic et al., 2005; Leclerc et al., 2019; Plailly et al., 2012), gustatory (Belardinelli et al., 2009; Kobayashi et al., 2004, 2011), tactile (Schmidt et al., 2014; Yoo et al., 2003), and motor imagery (Grèzes & Decety, 2000; Hanakawa et al., 2008; Hétu et al., 2013). Here, some studies showed a positive correlation between sensory cortex activation and imagery vividness (Belardinelli et al., 2009; Cui et al., 2007; Herholz et al., 2012), while others showed that the content of visual imagery can be decoded from visual cortex activity using multivariate decoding in fMRI (Albers et al., 2013; Koenig-Robert & Pearson, 2019; Naselaris et al., 2015). Similarly, low imagers rely less on visual cortex compared to high imagers when asked to complete visual imagery tasks (mental rotation; Logie et al., 2011), and studies using TMS show that engaging in visual imagery is associated with increased excitation in visual cortex (Cattaneo et al., 2011; Sparing et al., 2002). These findings, and others, linking imagery to activity in sensory cortices, lead us to propose that aphantasia may be characterised by low visual cortex excitation, and that this may in fact be part of a wider deficit across multiple cortices within the same brain. This may give rise to the multi-modal imagery impairments seen in people with aphantasia, consistent with our model in Figure 1.

It is important to recognise that there were rare instances where aphantasics reported *intact* imagery in non-visual senses (i.e., 3% of our aphantasics did *not* have absent/vague/dim imagery in another sense). Therefore, although having low imagery in one domain (visual) is often associated with imagery impairments in other senses, this may not *always* be the case. If our model holds true, having low cortical excitability in one sensory area may increase the likelihood of low excitability in other sensory areas, but we are proposing *likelihoods* not absolutes, and therefore expect individual differences. Testing these models with neuroscientific methods will be a fruitful avenue for future research. Alternatively, however, it is possible that even these 3% did have other imagery weaknesses, but simply in sense domains we did not test (e.g., interoception, thermoception, nocioception, etc.). Therefore, testing these domains will be an important avenue for future research.

A second feature of our model was the prediction that imagery may also link to sensory sensitivity (under- and over-responsiveness to sensory stimuli entering via the sense organs). In support of our model, we found that aphantasic individuals reported fewer sensory sensitivities, both overall (i.e., for both hypo- and hyper-sensitivity) and within every sense domain tested with the exception of audition. This effect remained even when factoring out autistic traits. In Experiment 2 we extended this finding to the general population, showing again a positive correlation between imagery and sensitivity, while again factoring out AQ scores. In Experiment 3, we provided behavioural support: aphantasic individuals demonstrated less sensitivity than controls in a *Pattern Glare Task* (i.e., fewer visual effects from high/medium gratings and less visual discomfort from high gratings). Overall, our findings suggest that imagery and sensory sensitivity are linked, potentially (our model suggests) via cortical excitability. We noted that cortical excitability has been traditionally associated with levels of *hyper-*sensitivity (i.e., increased cortical excitability linked to hyper-responsiveness; Green et al., 2015, 2013; Wilkins et al., 1984). However, hyper- and hypo-sensitivities are thought to be linked via *adaptation* (Takarae & Sweeney, 2017; Ward, 2019). This led us to predict that aphantasics – who we propose are *low* in cortical excitability – would show not only less hyper-sensitivity but also less hypo-sensitivity. This is indeed what we found. It is unclear why low sensitivity for aphantasics did not extend to auditory items (especially since sensory sensitivity is correlated across modalities). However, the auditory channel is also remarkable in being the most sensitive (Robertson & Simmons, 2013; and see Figure 5 for this clear difference). Further research is needed to better determine whether our null effect is genuine, by using alternative measures of auditory sensory sensitivity including those that employ sound stimuli and not simply questionnaire self-report.

What specific neural mechanisms might underpin low excitation across multiple sensory cortices in aphantasia? Our model fits broadly with neural noise theories from the autism literature which suggest that high levels of sensory sensitivity in people with autism are driven – at least in part – by increased levels of neural noise (excitation) within sensory cortices (Milne, 2011; Rubenstein & Merzenich, 2003; Simmons, 2019; Simmons et al., 2009). This theory links neural noise with an increase in excitatory (e.g., glutamate) synaptic activity and a reduction of inhibitory (e.g., GABA) synaptic activity (e.g., Rubenstein & Merzenich, 2003; Wood et al., 2021). Although speculative at this point, the mechanisms behind low cortical excitability in aphantasia may therefore include lower levels of excitatory glutamatergic synaptic activity, and/or higher inhibitory GABA-ergic synaptic activity in sensory cortices and areas involved in sensory modulation/regulation (e.g., thalamus; Wood et al., 2021). Depending on the sensory cortices affected by these patterns of excitation, this may give rise to lower imagery, and sensitivity, in many, several, or a single sense domain. As a parallel, we note that differences across disparate brain regions have also been found in a number of other sensory conditions, for example in synaesthesia where hyper-connectivity of white matter can arise in multiple brain regions, giving rise to different manifestations of what is considered the same underlying condition (Rouw & Scholte, 2007; Rouw et al., 2011).

An important question raised by our findings is how aphantasia and autism are related. People with aphantasia report *high* levels of autism traits (Dance et al., 2021), and autism is usually linked with heightened sensory sensitivity (Ben-Sasson et al., 2009; Bogdashina, 2003; Horder et al., 2014; Robertson & Simmons, 2013, 2015; Simmons et al., 2009). But here we found the opposite: aphantasics showing *lower* levels of sensitivity. Moreover, we still found that AQ scores were positively related to sensitivity in our participants overall (when included as a covariate in our analysis of sensory sensitivity in Experiment 1). A simple explanation may lie in the fact that autism is a cluster of traits in different domains, and some populations (e.g., aphantasics) may show higher scores in only certain domains. We know already that aphantasics show weak imagination and social skills, but match controls on other autism traits such as attention-to-detail, attention switching, and communication (Dance et al., 2021). In other words, aphantasics may be like people with autism in some ways (imagination, social skills) but not in others (attention-to-detail – and indeed sensory sensitivity). Similar patterns have been found for other conditions, such as people with synaesthesia, who have higher AQ scores because they share the autistic trait of (only) attention-to-detail (Van Leeuwen et al., 2019; Ward et al., 2018, 2017). It is possible that individuals with autism (or high in autistic traits) may experience somewhat less intense sensory sensitivity if they *also* have aphantasia. Teasing apart the specific instances where imagery maps directly onto sensory sensitivity will be an interesting avenue for future research. For now, we have begun to unravel the relationship between imagery and sensitivity by showing that aphantasic individuals experience lower sensory sensitivity than people who have visual imagery.

Finally, our study also provides answers about the very nature of aphantasia. Aphantasia was originally named for its links to the Greek word for imagination (φαντασία) and the related term φάντασμα (phántasma, “phantasm, an appearance, image, apparition, spectre” (Online Etymology Dictionary, 2001; Thomas, 2011; Zeman et al., 2015). It also has etymological links to the word φαντάζω (phantázō, “I make visible”; Online Etymology Dictionary, 2001) which make it a highly useful term to describe an absence of imagery that is visual per se. However, our finding here that aphantasia is part of a broader deficit in imagery (see also Dawes et al., 2020; Zeman et al., 2020) leads us to suggest a second complementary term, *dysikonesia*. Like aphantasia, we propose that dysikonesia encompasses imagery that is absent or dim/vague, but we introduce this term to describe the *broader* phenotype, i.e., imagery deficits across multiple domains (as found in 97% of aphantasics tested here), or indeed for cases where the particular domain of the imagery deficit has not been specified. Its etymological root (*icon*^2^) has the useful meaning of a form which reflects its referent, in the same way that visual imagery has qualities that reflect visual entities in the world, and auditory imagery has qualities that reflect auditory entities in the world, and so on. People with dysikonesia, we suggest, therefore have visual and auditory (and other sensory) knowledge, but without the iconic quality of imagery. This classification makes aphantasia one sub-class of dysikonesia, and 3% of our aphantasic group experienced this subclass only (i.e., imagery deficit in only vision). Overall, our results provide an important step forward in understanding the experience of imagery in aphantasic individuals, and we open up a wider debate about the phenomenology, and indeed the causes and consequences of absent imagery.

In conclusion, our study is the first to characterise the relationship between mental imagery and sensory sensitivity, the first to model this relationship, and the first to name and model a phenotype of poor imagery cross-senses. Our findings raise questions about the best way to define aphantasia, which we now embed within a broader multi-modal imagery deficit we term *dysikonesia*. We present both self-report and behavioural methods, and propose a model linking imagery and sensory sensitivity via neural excitability within sensory cortices.

## Supplemental Material

sj-docx-1-pec-10.1177_03010066211042186 - Supplemental material for What is the Link Between Mental Imagery and Sensory Sensitivity? Insights from Aphantasia Click here for additional data file. Supplemental material, sj-docx-1-pec-10.1177_03010066211042186 for What is the Link Between Mental Imagery and Sensory Sensitivity? Insights from Aphantasia by C. J. Dance, J. Ward and J. Simner in Perception

## Notes

1. This age difference of ~5 years across groups was significant (*t*(299.72) = −2.89, *p* = .004). However, all our analyses (see Results) remain the same when repeated with age as a covariate (see Supplementary Information).

2. In dysikonesia, we have chosen an earlier form of orthography (ikon) simply to disambiguate pronunciation (i.e., avoiding the ambiguity of /c/ = [k] or [s]).
