# Supplementary material for: What is the Link Between Mental Imagery and Sensory Sensitivity? Insights from Aphantasia
Source: Perception. 2021 Aug 31;50(9):757–82. doi: 10.1177/03010066211042186 (PMC8438787; doi:10.1177/03010066211042186)
Supplement: sj-docx-1-pec-10.1177_03010066211042186 - Supplemental material for What is the Link Between Mental Imagery and Sensory Sensitivity? Insights from Aphantasia [file sj-docx-1-pec-10.1177_03010066211042186.docx]

**Supplementary Information**

**Table S1.**

*Groups means in mental imagery (with standard deviations, SDs) for Aphantasics and Controls, in each of the sense domains. The table shows independent samples t-tests with Welch correction, with bootstrapped 95% confidence intervals (BCa bootstrapping performed 1000 times). Uncorrected p values are shown, with significant differences after correcting for multiple comparisons (using the Benjamini-Hochberg False Discovery Rate method; Benjamini & Hochberg, 1995, 2000) highlighted in bold (i.e., all survive).*

| Sense domain | Measure | Aphantasics | | Controls | | *t* | *df* | *p* | *g* | BCa 95% CIs | |
| --- | --- | --- | --- | --- | --- | --- | --- | --- | --- | --- | --- |
|  |  | *M* | *SD* | *M* | *SD* |  |  |  |  | Lower | Upper |
| Visual | Psi-Q | .45 | 1.18 | 7.70 | 2.02 | 37.25 | 212.11 | **<.001** | 4.48 | 6.87 | 7.66 |
| Auditory | CAIS | 24.66 | 16.31 | 61.73 | 12.60 | 22.26 | 297.89 | **<.001** | 2.51 | 33.74 | 40.34 |
|  | Psi-Q | 1.76 | 2.94 | 7.87 | 2.00 | 21.39 | 287.71 | **<.001** | 2.39 | 5.51 | 6.68 |
| Tactile | Betts-ad | 16.85 | 9.73 | 45.85 | 9.54 | 26.09 | 293.09 | **<.001** | 3.00 | 26.56 | 31.11 |
|  | Psi-Q | 1.18 | 2.43 | 7.81 | 2.00 | 26.01 | 299.87 | **<.001** | 2.95 | 6.14 | 7.13 |
| Olfactory | VOIQ | 20.82 | 11.31 | 55.96 | 13.60 | 24.13 | 266.85 | **<.001** | 2.83 | 32.30 | 37.69 |
|  | Psi-Q | .83 | 1.97 | 7.42 | 2.11 | 27.88 | 283.15 | **<.001** | 3.23 | 6.14 | 7.05 |
| Gustatory | Betts-ad | 15.61 | 8.41 | 45.52 | 9.34 | 29.00 | 278.73 | **<.001** | 3.37 | 27.75 | 31.98 |
|  | Psi-Q | .92 | 2.03 | 7.47 | 2.15 | 27.03 | 284.72 | **<.001** | 3.13 | 6.09 | 7.01 |
| Bodily | Betts-ad | 19.98 | 11.41 | 44.01 | 8.79 | 20.65 | 297.74 | **<.001** | 2.33 | 21.83 | 26.20 |
|  | Psi-Q | 1.34 | 2.36 | 7.51 | 1.98 | 24.67 | 300.00 | **<.001** | 2.80 | 5.69 | 6.66 |
| Movement | VMIQ | 17.27 | 9.93 | 44.12 | 10.97 | 22.13 | 279.42 | **<.001** | 2.57 | 24.64 | 29.13 |
| Feeling | Psi-Q | 2.57 | 3.43 | 7.56 | 1.82 | 16.14 | 256.10 | **<.001** | 1.77 | 4.29 | 5.65 |

|  |  |
| --- | --- |

**Table S2.**

*Groups means in sensory sensitivity (with standard deviations, SDs) for Aphantasics and Controls, in each of the sense domains (GSQ). The table shows independent samples t-tests with Welch correction, with bootstrapped 95% confidence intervals (BCa bootstrapping performed 1000 times). Uncorrected p values are shown, with significant differences after correcting for multiple comparisons (using the Benjamini-Hochberg False Discovery Rate method; Benjamini & Hochberg, 1995, 2000) highlighted in bold (i.e., all survive).*

| Sense domain | Aphantasics | | Controls | | *t* | *df* | *p* | *g* | BCa 95% CIs | |
| --- | --- | --- | --- | --- | --- | --- | --- | --- | --- | --- |
|  | *M* | *SD* | *M* | *SD* |  |  |  |  | Lower | Upper |
| Visual | 7.32 | 4.17 | 9.37 | 5.20 | 3.72 | 261.33 | **<.001** | 0.44 | 1.01 | 3.11 |
| Auditory | 11.95 | 4.63 | 12.09 | 4.65 | .265 | 290.88 | .791 | 0.03 | -.924 | 1.19 |
| Tactile | 6.96 | 4.12 | 8.84 | 5.13 | 3.46 | 261.48 | **.001** | 0.41 | .791 | 2.94 |
| Olfactory | 6.35 | 3.50 | 8.74 | 5.35 | 4.49 | 228.46 | **<.001** | 0.54 | 1.37 | 3.36 |
| Gustatory | 7.00 | 4.02 | 8.82 | 4.98 | 3.45 | 262.25 | **.001** | 0.41 | .698 | 2.86 |
| Proprioception | 5.22 | 3.54 | 7.57 | 5.75 | 4.18 | 219.52 | **<.001** | 0.50 | 1.25 | 3.45 |
| Vestibular | 5.72 | 3.81 | 8.20 | 5.60 | 4.41 | 234.64 | **<.001** | 0.52 | 1.46 | 3.57 |

**Results of Experiment 1 when including age as a covariate**

In the two sections below (re: imagery and sensory sensitivity), we repeat our analyses of Experiment 1, but include now a covariate of age. In our group sampling, we found an unintended groupwise difference in age (aphantasics: *M* = 42.35, *SD* = 15.95; controls *M* = 37.39, *SD* = 13.83; *t*(299.72) = -2.89, *p* = .004. To ensure this ̴5 year age gap did not influence our results, we repeat our analyses here, with age as a covariate. To anticipate our findings, our overall pattern of results remained unchanged, for both imagery and sensitivity.

*Do Aphantasics have Poor Imagery in Multiple Domains?*

To examine if age influenced our results for imagery, we replicated our main analysis for the Psi-Q, but this time including age as a covariate. We conducted a 2x7 ANCOVA crossing group (aphantasics, controls) with sense domain (Psi-Q imagery subscales; visual, auditory, olfactory, gustatory, tactile, body sensation, and feeling), adding age as a covariate. As before, there was a significant main effect of group (*F*(1, 299) = 834.51, *p* <.001, ﻿η_p_﻿^2^ = .736), a significant main effect of sense domain (*F*(3.93, 1174.08) = 7.85, *p* <.001, ﻿η_p_﻿^2^ = .026; with Greenhouse-Geisser correction), and a significant interaction between the two (*F*(3.93, 1174.08) = 21.35, *p* <.001, ﻿η_p_﻿^2^ = .067; with Greenhouse-Geisser correction. There was no significant effect of age (*F*(1, 299) = .035, *p* = .852, ﻿η_p_﻿^2^ = .000), and no interaction between age and sense domain (*F*(3.93, 1174.08) = 1.63, *p* = .165, ﻿η_p_﻿^2^ = .005; with Greenhouse-Geisser correction).

Overall, our results show that when controlling for the influence of age our pattern of results for imagery remain the same.

*Does Imagery Predict Sensory Sensitivity?*

Next, we repeated our sensory sensitivity (GSQ) analysis, by conducting a 2x2x7 ANCOVA crossing group (aphantasic, control) with sensitivity type (hyper-, hypo-sensitivity) and sense domain (GSQ subscales; visual, auditory, olfactory, tactile, proprioception, vestibular, gustatory). As before, we included AQ scores as a covariate to control for the influence of autism traits, and this time we included age as an additional covariate to control for the influence of participant age. The ANCOVA revealed a significant main effect of age (*F*(1, 298) = 18.08, *p* <.001, η_p_﻿^2^ = .057), indicating that overall sensory sensitivity reduced with increasing age. Despite this effect of age, our main pattern of results emerged as before. Replicating our main analysis, the ANCOVA revealed a significant main effect of group, (*F*(1, 298) = 20.78, *p* <.001, ﻿η_p_﻿^2^ = .065), a significant interaction between group and sensitivity-type (hyper/hypo), (*F*(1, 298) = 6.32, *p* = .012, ﻿η_p_﻿^2^ = .021; with Greenhouse-Geisser correction), and a significant interaction between group and sense domain, (*F*(5.16, 1538.58) = 5.96, *p* <.001, ﻿η_p_﻿^2^ = .020; Greenhouse-Geisser correction). This time, there was also a significant main effect of sensitivity type, (*F*(1, 298) = 8.55, *p* = .004, η_p_﻿^2^ = .028; with Greenhouse-Geisser correction), reflecting that overall participants tended to report more hyper-sensitivities than hypo-sensitivities. As before, the ANCOVA also revealed a number of other effects which were unrelated to our hypotheses (which again we did not explore further to reduce proliferation of multiple comparisons). For example, there was a significant main effect of AQ score, (*F*(1, 298) = 89.88, *p* <.001, η_p_﻿^2^ = .232), and a significant effect of sense domain, (*F*(5.16, 1538.58) = 3.34, *p* = .005, η_p_﻿^2^ = .011; Greenhouse-Geisser correction).

In sum, our results show that when controlling for the influence of age, the overall pattern of results for both our analyses (imagery and sensitivity) remain unchanged.
